# Predicting *N*-Strain Coexistence from Co-colonization Interactions: Epidemiology Meets Ecology and the Replicator Equation

**DOI:** 10.1007/s11538-020-00816-w

**Published:** 2020-10-29

**Authors:** Sten Madec, Erida Gjini

**Affiliations:** 1 Institut Denis Poisson, University of Tours, Tours, France; 2 Instituto Gulbenkian de Ciência, Oeiras, Portugal; 3 Present Address: Center for Computational and Stochastic Mathematics, Instituto Superior Técnico, University of Lisbon, Lisbon, Portugal

**Keywords:** Multi-strain SIS model, Coinfection, Slow-fast dynamics, Weak selection, Competition-cooperation, Multispecies coexistence, Invasion fitness network

## Abstract

Multi-type infection processes are ubiquitous in ecology, epidemiology and social systems, but remain hard to analyze and to understand on a fundamental level. Here, we study a multi-strain susceptible-infected-susceptible model with coinfection. A host already colonized by one strain can become more or less vulnerable to co-colonization by a second strain, as a result of facilitating or competitive interactions between the two. Fitness differences between *N* strains are mediated through $N^2$ altered susceptibilities to secondary infection that depend on colonizer-cocolonizer identities ($K_{ij}$). By assuming strain similarity in such pairwise traits, we derive a model reduction for the endemic system using separation of timescales. This ‘quasi-neutrality’ in trait space sets a fast timescale where all strains interact neutrally, and a slow timescale where selective dynamics unfold. We find that these slow dynamics are governed by the replicator equation for *N* strains. Our framework allows to build the community dynamics bottom-up from only pairwise invasion fitnesses between members. We highlight that mean fitness of the multi-strain network, changes with their individual dynamics, acts equally upon each type, and is a key indicator of system resistance to invasion. By uncovering the link between *N*-strain epidemiological coexistence and the replicator equation, we show that the ecology of co-colonization relates to Fisher’s fundamental theorem and to Lotka-Volterra systems. Besides efficient computation and complexity reduction for any system size, these results open new perspectives into high-dimensional community ecology, detection of species interactions, and evolution of biodiversity.

**Electronic supplementary material:**

The online version of this article (10.1007/s11538-020-00816-w) contains supplementary material, which is available to authorized users.

## Introduction

One of the fundamental questions in ecology and evolutionary biology is the generation and maintenance of biodiversity (Gause 1934; MacArthur 1967; Tilman 1987; Hubbell 2001). Many theoretical approaches consider multi-species interactions with classical Lotka-Volterra systems (Pascual and Dunne 2006; Mougi and Kondoh 2012) or with evolutionary game theory models (Nowak and May 1992; Traulsen and Nowak 2006), where the interplay between cooperation and competition is key. Typically where population structure is involved, for example in multi-strain infectious disease epidemiology (Kucharski et al. 2016), and in high-dimensional spaces of diversity, analysis can become prohibitive, and computational simulations are often adopted instead (Cobey and Lipsitch 2012a; Bottomley et al. 2013; Nurhonen et al. 2013).

However, several analytical advances have been made to study antigenic diversity and coexistence in multi-strain pathogens. These methods were based on simplifying high-dimensional SIR models with strain-specific and cross-immunity interactions (Gog and Grenfell 2002; Gupta and Anderson 1999; Lin et al. 1999; Ferguson et al. 1999); prominent features of immunizing infections such as influenza, dengue or malaria. In contrast, diversity and interactions arising among strains through co-colonization (or co-infection), implying simultaneous carriage of two or more strains, have received less mathematical attention on the SIS modeling spectrum (Adler and Brunet 1991). Theoretical multi-strain SIS models have advanced analytically by either neglecting coinfection (Martcheva 2009), modeling typically only 2 strains when incorporating this process (Lipsitch 1997; Gjini et al. 2016; Gjini and Madec 2017), or collapsing strain interactions to a single mean-field parameter to focus on other traits, e.g., virulence (van Baalen and Sabelis 1995; Mosquera and Adler 1998; Alizon 2013a). More recently, coinfection SIS dynamics between 2 and 3 interacting diseases, under the additional effects of host contact structure, have been modeled (Hébert-Dufresne and Althouse 2015; Chen et al. 2017; Pinotti et al. 2019), uncovering strong effects of cooperation between diseases. Thus, SIS coinfection models under the interplay of cooperative and competitive interactions, among an arbitrary number of interacting entities, remain undeveloped. Consequently, our understanding of how such interactions may promote realistic coexistence processes within and between microbial species (Bogaert et al. 2011; Dunne et al. 2013; Shrestha et al. 2013; Cohen et al. 2008; Abdullah et al. 2017), and how they may link with empirical data (Lipsitch et al. 2012) and interventions (Weinberger et al. 2011), remains limited.

In this paper, we address this gap. Inspired by the epidemiology of multi-strain SIS infectious diseases, we develop a conceptual framework for thinking about co-colonization (co-infection) in high-dimensional interaction space. To increase our understanding of coexistence in such multi-type interacting systems, there is a need to expand our analytical power over a larger and more realistic resolution of diversity (higher number of interacting entities *N*). There is a need to study cooperation and competition under the same framework. Finally, there is a need to simplify the mathematics underlying such systems, in order to enable key biological principles and features to emerge, before adding more complexity or diversity layers. Here, we provide a fundamental advance on these three fronts. We model co-colonization interactions as a route to coexistence. We uncover an analytically tractable model reduction for an SIS system with *N* similar strains interacting in co-colonization. We show how this method enhances our understanding of cooperation and competition within the same formalism, exposing the role of mean-field values as well as variation around the mean for multi-strain dynamics.

In particular, we show that by assuming small differences in altered susceptibilities to co-colonization between *N* types, an explicit reduced system emerges via a timescale separation. This decomposes the total dynamics into a fast (neutral) and a slow (non-neutral) component, driven by variation in co-colonization traits. Thus, we extend the approach presented for $N=2$ (Gjini and Madec 2017). We derive an analytic solution for *N* strain frequencies over long time in a changing fitness landscape, which corresponds to the replicator equation from game theory (Taylor and Jonker 1978; Weibull 1997; Hofbauer and Sigmund 2003). Multi-strain epidemiological competition can be very complex, but our model reduction helps to simplify these dynamics, increases predictability, and highlights the role of key parameters for linking between coinfection epidemiology and eco-evolutionary feedbacks (Lion 2018). When written in terms of pairwise invasion fitnesses between strains, this model provides quantitative insights into system resilience to invasion and relates explicitly with adaptive dynamics (Metz et al. 1992).

Although the system is treated in an epidemiological spirit,^1^ parallels and conceptual analogies with other contexts can be easily drawn, where *N* similar types (species/strains/propagules), in a homogeneous mixing scenario, compete for free and singly occupied niches via generic colonizer-cocolonizer interactions. Co-colonization processes appear in many diverse ecological communities, from plant and marine ecosystems to infectious diseases: two species encountering and interacting locally in a unit of space or resource. How each entity, when colonizer, alters the local ‘environment’ for a second co-colonizer entity, is the abstract phenomenon modeled here; this could exhibit asymmetries, randomness, and particular numerical structures, ranging from facilitation to competition. Special cases for low dimensionality may be tractable analytically, but the entangled network that arises between $N^2$ such interacting pairs in co-colonization encounters, and its consequences for global *N*-type coexistence remain elusive. Here, we shed light on how the net behavior of such a system with multiple members emerges from pairwise outcomes in co-colonization. Our framework and findings should contribute both to a fundamental question about the structure of systems and to a significant mathematical challenge.

The paper is organized as follows. In Sect. 2, we present the model. In Sect. 3.1, we outline the key technical steps leading to the slow-fast decomposition for the $N-$strain system. In Sect. 3.2, we provide an equivalent representation in terms of the pairwise invasion fitness network between strains, and highlight the key quantities of our replicator equation. In Sect. 3.3, we show how this model reduction can be used to understand collective $N-$strain dynamics, by illustrating special interaction structures among strains in the system. In Sect. 3.4, we link the strain frequencies back to the epidemiological variables, highlighting emergent principles and the validity of our approximation.

## The Model: *N*-Strain SIS Dynamics with Co-colonization

**Fig. 1:**
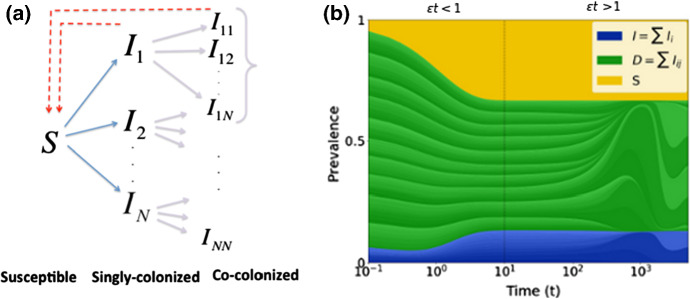
Model summary diagram. **a** Co-colonization model structure. Hosts move from susceptible to singly colonized state, and from singly colonized to co-colonized state. Clearance happens at equal rate for single and co-colonization. Co-colonization rate by strain *j* of singly colonized hosts with *i* is altered by a factor $K_{ij}$ relative to uncolonized hosts. There are $1+N+N^2$ states for hosts in the system. **b** Complex epidemiological dynamics can be represented in two interrelated timescales. Assuming that pairwise interaction coefficients in co-colonization can be written as: $K_{ij}=k+\varepsilon \alpha _{ij}$, the global compartmental dynamics can be decomposed into a fast and slow component. On the fast time-scale ($o(1/\varepsilon )$), strains follow neutral dynamics, driven by mean-field parameters, where total prevalence of susceptibles *S*, singly infected hosts, *I* and dually infected hosts, *D*, are conserved. On a slow time-scale, $\varepsilon t$, complex non-neutral dynamics between strains takes place, depicted here by the constituent variations within the blue and green. These non-neutral dynamics are here explicitly derived, and yield an explicit closed equation for strain frequency dynamics, reducing the model from $O(N^2)$ to *N* dimensions (Color figure online)

We consider a multi-strain infectious agent, transmitted via direct contact, following susceptible-infected-susceptible (SIS) dynamics, with the possibility of simultaneous colonization by two strains (coinfection). The model follows the structure of our previous study (Gjini and Madec 2017), but here the number of strains is *N*. With a set of ordinary differential equations (Fig.1a), we describe the proportion of hosts in several compartments: susceptibles, *S*, hosts colonized by one strain $I_i$, and co-colonized hosts $I_{ij}$ that carry strains from two colonization episodes. Among co-colonized hosts, our model includes also dually-infected hosts with the same strain, $I_{ii}$, and $I_{jj}$, as in Gjini et al. (2016); Gjini and Madec (2017), motivated by previous theoretical and evolutionary studies arguing for an unbiased relative fitness structure between any two strains (van Baalen and Sabelis 1995; Alizon 2013a), although there are other models that omit this class (Adler and Brunet 1991; Lipsitch 1997). Clearance rate $\gamma$ is assumed equal for single- and co-colonization episodes. This may be seen to reflect strain-transcending immunity, or other mechanisms of within-host growth limitation, that bring hosts back to the susceptible state without making them immune. Recruitment rate of susceptible hosts, *r*, equals the mortality rate from all compartments ($r=d$), so that total population size is constant. The parameter *r* may also reflect the per capita growth rate of the host, which is the same for all hosts, with all newborn hosts susceptible. We have:

1 $$\begin{aligned} {\left\{ \begin{array}{ll} {\dot{S}}=r -S{\displaystyle \sum _{j=1}^N F_j} - dS +\gamma (1-S),\\ \dot{I_i}=F_i S- I_i {\displaystyle \sum _{j=1}^N} K_{ij}F_j -(d+\gamma ) I_i,\quad 1\le i\le N \\ \dot{I_{ij}}=I_i K_{ij}F_j-(d+\gamma ) I_{ij} , \qquad 1\le i,j\le N\\ \end{array}\right. }\nonumber \\ \end{aligned}$$

where $F_{i}=\beta \left( I_i+\sum _{j=1}^N \frac{1}{2}(I_{ij}+I_{ji})\right)$ gives the force of infection of strain *i*. We assume that hosts colonized with two strains *i* and *j*, $I_{ij}$, transmit either with equal probability. Further, we assume equivalence in transmission $\beta$ and clearance rate $\gamma$, between strains, similar to previous formulations for $N\!=\!2$  (Gjini et al. 2016; Gjini and Madec 2017). All infected hosts have the same rate of total transmission to susceptible hosts, so from co-infected hosts, each type is transmitted at half the rate it would be from a singly infected host (and type does not affect transmission rate). Note that this implicitly assumes a weak interaction at the within-host level, because if the strains were transmitting independent of each other, co-colonized hosts would transmit each strain at exactly the same rate as the corresponding singly colonized hosts.

Strain diversity is manifested only in how current colonization modulates acquisition of a second strain. Any two strains interact via co-colonization coefficients $K_{ij}$, which denote relative factors of altered susceptibilities to host co-colonization by strain *j* when already colonized by *i*. Thus, $K_{ij}>1$ indicates that prior colonization with *i* facilitates co-colonization with *j*, while $K_{ij}<1$ describes that prior colonization with *i* hampers co-colonization by *j*. For more details on the parameters see Table S1.

Since we model *N* closely-related entities, we can write each co-colonization coefficient as: $K_{ij}=k+\varepsilon \alpha _{ij}$, where $0 \le \varepsilon<<1$ (Gjini and Madec 2017). When $\varepsilon =0$, $K_{ij}=k$ for all strain pairs, thereby leading to a system with identical strains in how they interact upon co-colonization. When $\varepsilon >0$, *k* reflects a suitable common reference (benchmark for all strains), and $\alpha _{ij}$, which can be positive or negative, represent deviations from that reference for all strain pairs. This will form the basis of our model reduction framework, and system decomposition into smaller sub-systems.

We use separation of time-scales, to understand global system dynamics (Fig.1b). It is not clear a priori neither which variables are the fast ones and the slow ones in our system, nor how they change over time. Substituting $K_{ij}$ by $k+\varepsilon \alpha _{ij}$ in (1) and re-arranging leads to an explicit system formulation with $\varepsilon$. After a series of mathematical manipulations, we obtain a tractable slow-fast formulation of the dynamics (see Supplementary Material 2 for details). We then analyze the case of $\varepsilon =0$, leading to perfect symmetry between strains in co-colonization ($K_{ij} \equiv k$), and the case of $\varepsilon >0$, describing realistic deviations from neutrality.

## Results

### From Epidemiology to Strain Frequencies with Slow-Fast Dynamics

#### Fast Timescale: Neutral Dynamics and Balancing of Aggregated Variables

In order to obtain a simpler representation for such a general *N* strain system, we use the following aggregation of variables:

2 $$\begin{aligned} J_i= I_i+\frac{1}{2}\sum _{j=1}^N (I_{ij}+I_{ji}), \quad I=\sum _{i=1}^N I_i ,\quad D= \sum _{i=1}^{N}\sum _{j=1}^N I_{ij},\quad \text {and }T=I+D, \end{aligned}$$

for the fraction of hosts transmitting strain *i* in the population, and the prevalence of single, double and overall colonization, respectively. Total prevalence satisfies $T=\sum _{i} J_i$ and the forces of infection are: $F_i=\beta J_i$. With the notations (2), and denoting $m=\gamma +r{=}\gamma +d$ (clearance+natural mortality), the system (1) can be rewritten as:

3 $$\begin{aligned}&{\left\{ \begin{array}{ll} {\dot{S}}=m(1-S)-\beta ST,\\ {\dot{T}}=\beta ST-mT,\\ \end{array}\right. } \end{aligned}$$

4 $$\begin{aligned}&{\left\{ \begin{array}{ll} \dot{I_i}=\beta J_i S-mI_i- \beta I_i \displaystyle \sum _{j=1}^N K_{ij}J_j \\ \dot{J_i}=(\beta S-m) J_i+\frac{\beta }{2} \displaystyle \sum _{j=1}^N {\left( K_{ji} J_i I_j- K_{ij} J_j I_i\right) },\\ \end{array}\right. },\quad 1\le i\le N,\nonumber \\ \end{aligned}$$

5 $$\begin{aligned}&\quad \!\dot{I_{ij}}=I_i K_{ij}\beta J_j -m I_{ij}, \qquad 1\le i,j\le N \end{aligned}$$

This system of $2+{2}N+N^2$ equations, now displays a convenient structure, that we exploit for our analysis: i) First, we describe the block (3) of 2 equations (*S*, *T*) that do not depend on $K_{ij}$. ii) Next, we study the block (4) of 2*N* equations $(I_i,J_i)$, which is the most complicated. iii) Lastly, we deal with the block (5) of the $N^2$ equations of $I_{ij}$, which is simple once the dynamics of $I_i$ and $J_i$ are known (see Supplementary Material 2 for details). Clearly, if the basic reproduction number $R_0=\frac{\beta }{m}>1$, then there is an endemic equilibrium, whereby $(S,T) \rightarrow \left( \frac{1}{R_0},1-\frac{1}{R_0}\right)$ (Dietz 1993). $R_0$ denotes how many new colonization episodes a typical colonized host causes in a totally susceptible population, over their entire infectious period; in our case $R_0$ is equal for all strains. We thus reduce the system to the invariant manifold $(S,T)=(S^{*},T^{*}) \equiv \left( \frac{1}{R_0},1-\frac{1}{R_0}\right)$. It assumed that *S* and *T* have already reached their steady-state values $S^{*}$ and $T^{*}$. Once it is known that $(I_i,J_j)\rightarrow (I_i^*,J_j^*)$, then the $N^2$ equations for co-colonization compartments imply $I_{ij}\rightarrow \frac{\beta }{m} I_i^* K_{ij} J_j^*.$ Thus, once the dynamics of the second set of equations are explicit, so are the dynamics of co-colonization variables, and ultimately of the entire system.

Based on the strain similarity principle,which assumes similar coefficients in co-colonization interactions between strains, we write: $K_{ij}=k+\varepsilon \alpha _{ij}$, where $0 \le \varepsilon<<1$. Replacing these in (4), and re-arranging, we obtain:

6 $$\begin{aligned} {\left\{ \begin{array}{ll} {\dot{I}}=m({T^*}-I)-\beta k {T^*}I -\varepsilon \beta \displaystyle \sum _{i=1}^N\sum _{j=1}^N I_i\alpha _{ij}J_j \\ \dot{I_i}=m(J_i-I_i) -\beta k{T^*}I_i-\varepsilon \beta I_i \displaystyle \sum _{j=1}^N \alpha _{ij}J_j \\ \dot{J_i}=\frac{\beta k}{2} \left( I J_i-I_i {T^*}\right) +\frac{\varepsilon \beta }{2} \displaystyle \sum _{j=1}^N \left( I_j \alpha _{ji} J_i-I_i\alpha _{ij} J_j\right) \\ \end{array}\right. },\nonumber \\ \end{aligned}$$

where $$1\le i\le N$$, and $$I =\sum {I_j}.$$

#### Neutral System

If $\varepsilon =0$, then we obtain the *Neutral model*, where all strain co-colonization coefficients are equal ($K_{ij}\equiv k$). The first equation in system (6) gives the time dynamics of single colonization prevalence in the system

$$\begin{aligned} I(t)= {I^*}+e^{-t(m+\beta k {T^*})} (I(0)-{I^*})\rightarrow {I^*}:=\frac{m {T^*}}{m+\beta k {T^*}}. \end{aligned}$$

Co-colonization prevalence is simply derived as: ${D^*}={T^*}-{I^*}$. Next, fixing $I={I^*}$, yields the *N* uncoupled linear systems:

7 $$\begin{aligned} \begin{pmatrix}\dot{I_i}\\ \dot{J_i}\end{pmatrix}=\begin{pmatrix}-(m+\beta k {T^*})&{}m\\ frac{\beta k {T^*}}{2} &{}\frac{\beta k {I^*}}{2}\end{pmatrix} \begin{pmatrix}{I_i}\\ {J_i}\end{pmatrix}=A_0\begin{pmatrix}{I_i}\\ {J_i}\end{pmatrix}. \end{aligned}$$

Matrix $A_0$ has the two eigenvalues 0 and $-\xi =tr(A_0)<0$. By defining $H_i$ and $z_i$ from the eigenvectors of $A_0$ as:

$$\begin{aligned} \begin{pmatrix} H_i\\ z_i\end{pmatrix}= \begin{pmatrix} 2{T^*}&{}{I^*}\\ {D^*}&{}{T^*}\end{pmatrix}^{-1} \begin{pmatrix} I_i\\ J_i\end{pmatrix}, \end{aligned}$$

that is

8 $$\begin{aligned} H_i=\frac{{I^*}{T^*}}{2({T^*})^2-{D^*}{I^*}} \left[ \frac{I_i}{{I^*}}-\frac{J_i}{{T^*}}\right] \text { and }z_i=\left( \frac{I_i}{{I^*}}\right) +\frac{2({T^*})^2}{2({T^*})^2-{D^*}{I^*}}\left( \frac{J_i}{{T^*}}-\frac{I_i}{{I^*}}\right) ,\nonumber \\ \end{aligned}$$

we have $\dot{H_i}=-\xi H_i$ and $\dot{z_i}=0.$ Thus on the fast time-scale $H_i\rightarrow 0$ and $z_i$ remains constant. The quantity $H_i$ measures the difference between the part occupied by strain *i* in single colonization ($\frac{I_i}{{I^*}}$) versus the part of strain *i* in total carriage ($\frac{J_i}{{T^*}}$). Thus, $H_i\rightarrow 0$ means that, on the fast timescale, the proportion of strain *i* in single colonization, tends to equalize the proportion of strain *i* in overall colonization ($\frac{I_i}{{I^*}} - \frac{J_i}{{T^*}} \rightarrow 0$). This implies it also tends to be equal to the proportion occupied by strain *i* in co-colonization: $\sum _{j \ne i} I_{ij}/2 + I_{ii}=D_i/{D^*}$. Because $H_i=0$ is the only equilibrium, we infer that after the fast dynamics $z_i$ may be expressed as:

9 $$\begin{aligned} z_i=\frac{J_i}{{T^*}}=\frac{I_i}{{I^*}}=\frac{D_i}{{D^*}}. \end{aligned}$$

Although it is not clear whether to use $J_i/T$, $I_i/I$, or $D_i/D$ to define strain frequencies, as time progresses, these definitions limit to the same quantity $z_i$, with $\sum z_i =1$.

#### Slow Dynamics: Emergence of the Replicator Equation for Strain Frequencies

Now, that we have $z_i$ with a clear meaning, in terms of strain frequencies, we can analyze the system for $\varepsilon >0$ (See Supplementary Material 3). We find that on the slow timescale $\tau =\varepsilon t$, strain frequencies, $z_i$, obey explicit dynamics on ${\mathcal {P}}=\{(z_i)_i \in [0,1]^N,\;\sum _{i=1}^Nz_i=1\}$:

10 $$\begin{aligned} \frac{d}{d\tau }z_i=\Theta z_i\left( \sum _{j=1}^N \left[ \mu (\alpha _{ji}-\alpha _{ij})+\alpha _{ji}\right] z_j -q(\mathbf{z })\right) , \quad 1\le i\le N, \end{aligned}$$

where the constants $\Theta , \mu >0$ are explicit functions of the global steady state (${T^*}, {I^*},{D^*}$) of the neutral model:

$$\begin{aligned} \Theta =\frac{\beta {T^*}{I^*}{D^*}}{2({T^*})^2-{I^*}{D^*}}\; \text {; }\; \mu =\frac{{I^*}}{{D^*}}=\frac{1}{k(R_0-1)}, \end{aligned}$$

and $q(\mathbf{z })$ is a quadratic term given by:

$$\begin{aligned} q(\mathbf{z })=\mathop {\sum \sum }_{1\le \kappa , j\le N} \alpha _{\kappa j}z_{\kappa }z_j. \end{aligned}$$

Equation 10 is a replicator equation for *N* strains (Taylor and Jonker 1978; Weibull 1997; Hofbauer and Sigmund 2003). By denoting $$A=\begin{pmatrix} \alpha _{ij} \end{pmatrix}_{1\le i,j \le N}$$ and $M=\mu (A^T-A)+A^T$, and using the fact that $\mathbf{z }^T(A^T-A)\mathbf{z }=0$ we have $q(\mathbf{z })=\mathbf{z }^T A\mathbf{z }=\mathbf{z }^T M \mathbf{z }$ so that the equivalence becomes clearer:

11 $$\begin{aligned} \frac{d}{d\tau }z_i=\Theta z_i\left( (M \mathbf{z })_i-\mathbf{z }^T M \mathbf{z }\right) ,\quad 1\le i\le N. \end{aligned}$$

At this stage, we can apply quasi-stationarity methods (Tikhonov 1952; Lobry and Sari 1998, 2005; Hoppensteadt 1966) to show that the solution of the full system tends to the solution of the slow-fast representation as $\varepsilon \rightarrow 0$.

Equation 10 shows how the ultimate competition between *N* strains is driven by asymmetries in co-colonization interactions (the $\alpha$’s), as well as by average quantities, such as $R_0$ and *k*, appearing within $\Theta$ and $\mu$. With this expression, the strain selection occurring in the slow time scale, becomes entirely explicit. A summary of key model quantities in terms of mean field parameters $R_0$ and *k* is given in Table 1.

In the frequency equation for each strain (10), there is a common term $q(\mathbf{z })$, which changes over time. This term represents the evolving impact of all the strains on their ‘common environment’, which in turn modifies their own fitness landscape. A more explicit way to interpret $q(\mathbf{z })$ is in terms of relative change in ‘effective’ mean interaction coefficient between all extant types in the system, which if negative, indicates a global trend toward more pairwise inhibition in co-colonization coefficients, and if positive, indicates a global trend toward more pairwise facilitation. Formally in our system, we obtain this mean co-colonization trait dynamics as:

$$\begin{aligned} {\bar{k}}_{{\text {effective}}}(t)=k+\varepsilon q(\mathbf{z }). \end{aligned}$$

**Table 1:** Key model quantities in terms of basic reproduction number $R_0$ and reference interaction coefficient in co-colonization *k*

Symbol	Interpretation	Features
$R_0$	Basic reproduction number	$R_0=\beta /(\gamma +r) >1$
*k*	Reference interaction coefficient between types in co-colonization (e.g., mean of $K_{ij}$)	$$k{\left\{ \begin{array}{ll} <1: \text {competition},\\ >1: \text {cooperation}\end{array}\right. }$$
${S^*}$	Equilibrium prevalence of susceptibles	${S^*}=\frac{1}{R_0}$
${I^*}$	Equilibrium prevalence of singly colonized hosts	${I^*}=\frac{R_0-1}{R_0[1+k(R_0-1)]}$
${D^*}$	Equilibrium prevalence of co-colonized hosts	${D^*}=\frac{(R_0-1)^2 k}{R_0[1+k(R_0-1)]}$
$\mu$	Ratio between single and co-colonization	$\mu =\frac{{I^*}}{{D^*}}=\frac{1}{(R_0-1)k}$
$\Theta$	Rate of slow dynamics for strain frequencies	$\Theta =\beta \bigg (1-\frac{1}{R_0}\bigg )\bigg ( \frac{\mu }{2(\mu +1)^2-\mu }\bigg )$
$I_{ij}$	Co-colonization prevalence with *i* and *j*	$I_{ij}=kR_0[1+k(R_0-1)] I_i I_j$

### Strain Frequency Dynamics in Terms of Pairwise Invasion Fitnesses

Rewriting the replicator equation in terms of pairwise invasion fitnesses (Metz et al. 1992; Geritz et al. 1998; Meszéna et al. 2005), we uncover an equivalent very useful representation of the biological game dynamics among strains. Let $\lambda _i^j$ be the exponential growth rate of strain *i* evaluated when introduced at the trivial endemic equilibrium of the strain *j* alone. If the fitness $\lambda _i^j>0$, strain *i* will invade *j*, and viceversa, if $\lambda _i^j<0$, strain *i* cannot invade *j*. By considering the rate of growth of strain *i* in an endemic equilibrium set by *j* ($\frac{d}{d\tau }z_i$ in Eq. (10) in the special case where all $z_{\kappa }=0$ for ${\kappa \ne j}$), we find the exact formulation of pairwise invasion fitness, in our model, is given by:

12 $$\begin{aligned} \lambda _i^j=\alpha _{ji}-\alpha _{jj} +\mu (\alpha _{ji}-\alpha _{ij}). \end{aligned}$$

The first term in the invasion fitness quantifies the extent to which strain *j* facilitates strain *i* in co-colonization, and adds to the invasion fitness for strain *i*. The second term in the invasion fitness quantifies the extent to which resident strain *j* facilitates itself, and detracts from the fitness of invader strain *i*. The last term in the invasion fitness has to do with transitions from pure single colonization $I_j$ (resident) to mixed co-colonization $I_{ij}$ (resident + invader). Recall that once in the mixed co-colonization compartment $I_{ij}$, either strain can be transmitted with equal probability. The difference $(\alpha _{ji}-\alpha _{ij})$ quantifies the extent to which strain *j* facilitates strain *i* ($\alpha _{ji}$), and the extent to which strain *i* facilitates strain *j* ($\alpha _{ij}$). The relative benefit of *i* from this effect will be amplified with higher opportunity for co-colonization in the system (i.e., higher $\mu =I^*/D^*)$. Notice that $\alpha _{ii}$ does not appear in the invasion fitness of *i* because by definition, strain *i* is initially rare.

After some algebra (see Supplementary Material 3), the strain frequency dynamics of system (10) can be recast in terms of invasion fitnesses:

13 $$\begin{aligned} \frac{d}{d\tau } z_i = \Theta z_i \cdot \left( \sum _{j\ne i} \lambda _i^j z_j -\mathop {\sum \sum }_{1\le \kappa \ne j\le N} \lambda _j^{\kappa }z_jz_{\kappa } \right) . \end{aligned}$$

Ultimately, it is this matrix $\Lambda {=(\lambda _i^j)_{1\le i,j\le N}}$ that defines all ‘edges’ of the rescaled interaction network between *N* strains. Each edge corresponds to a sub-system with $N=2$, whose dynamics we have analyzed in detail in Gjini and Madec (2017). For $\lambda _1^2\lambda _2^1\ne 0$, in the $N=2$ co-colonization model, there are only four possible outcomes between 2 strains (edge linking 1 and 2): **i)** $\underline{\lambda _1^2>0 ,\lambda _2^1 >0:}$ stable coexistence of 1 and 2; **ii)** $\underline{\lambda _1^2<0 , \lambda _2^1 <0 }$: bistability of 1-only and 2-only, also known as a priority effect; **iii)** $\underline{\lambda _1^2 >0 , \lambda _2^1 <0 }$: 1-only competitive exclusion; **iv)** $\underline{\lambda _1^2 <0 , \lambda _2^1 >0 }$: 2-only competitive exclusion; similar to the classical competitive Lotka-Volterra model (Lotka 1926; Volterra 1926). Now, by knowing all pairwise invasion fitnesses between each couple of strains, via expression (13) we can reconstitute the ultimate dynamics of the full system with *N* types and co-colonization. The pairwise traits in the emergent expression are related to the original co-colonization traits, but now capture the essence of competition dynamics between different strains; invasion fitness (Metz et al. 1992; Geritz et al. 1998; Meszéna et al. 2005).

The entire dynamics of the *N*-strain ‘game’ can now be recapitulated based only on knowledge of the pairwise invasion network between each two ‘players’, allowing for bottom-up understanding. The global fitness of each strain in the system, depends not only on its own individual fitness and frequency at any time, but also on the fitnesses and frequencies of all other strains. Such interdependence can lead to a multitude of outcomes, as already recognized in replicator equation studies (Nowak and Sigmund 2004; Cressman and Tao 2014).

**Fig. 2:**
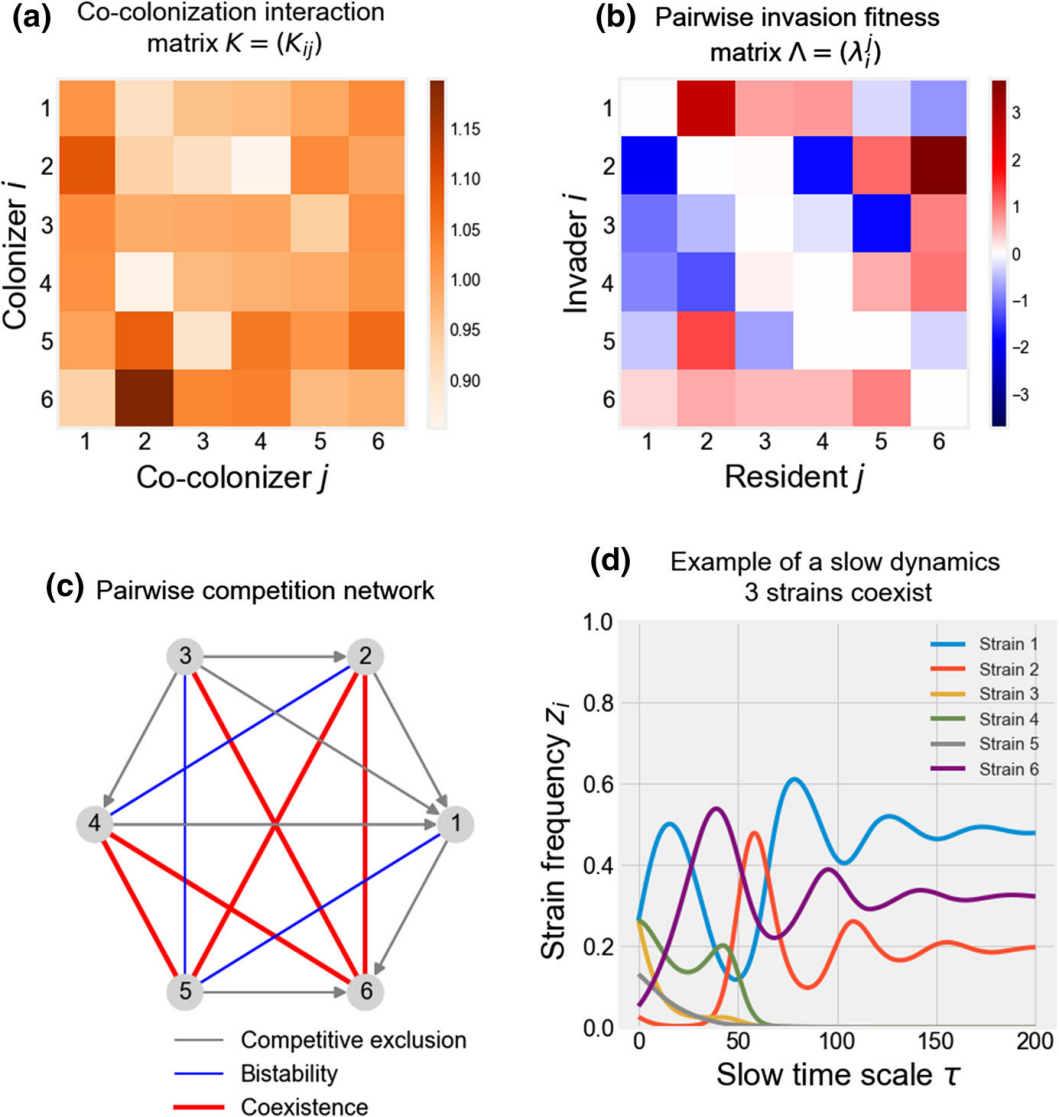
Example dynamics of our model for $N=6$. **a** The matrix of interaction coefficients in co-colonization (*K*), generated randomly, with mean $k=1$, and standard deviation $\varepsilon =0.1$. **b** The corresponding pairwise invasion fitness matrix ($\Lambda$) has been computed and visualized for assumed $R_0=2$. **c** The multi-strain network where each edge displays the outcome of pairwise invasion between any couple of strains, and the direction of grey edges denotes the winner in competitive exclusion. **d** Slow frequency dynamics resulting from these qualitative and quantitative interactions among entities (Eq. 13). A dynamic display of the trajectory is shown in Supplementary Movie S1 (Color figure online)

For illustration, in Fig.  2 and in Supplementary Movie S1, we provide an example of our modeling framework and the coexistence dynamics that arise among a number of strains (here $N=6$) for an arbitrary co-colonization interaction matrix *K*. In order to distinguish between the two levels of interaction among strains, we use the terms *colonizer and co-colonizer* strain when referring to $K_{ij}$, i.e., co-colonization interactions at the single host level, and the terms *resident and invader* strain when referring to $\lambda _i^j$ in pairwise invasion at the epidemiological level. Another combination of parameters, leading to a limit cycle for $N=6$, is illustrated in Supplementary Figure S1 and Supplementary Movie S2. These examples demonstrate that even weak asymmetries between apparently similar types, in altered susceptibilities to co-colonization, have the potential to generate rich and hierarchical collective behavior over long time.

#### Key Quantities of this Replicator Equation: $\Theta$, $\mu$ and Mean Fitness *Q*

In compact form, our *N*-strain frequency dynamics (13) can be written as:

$$\begin{aligned} \frac{d}{d\tau } \mathbf{z }_i = \Theta \mathbf{z }_i \cdot \big ((\Lambda \mathbf{z })_i -\mathbf{z }^t \Lambda \mathbf{z }\big ),\quad \quad i=1,\cdots ,N, \end{aligned}$$

by denoting the pairwise invasion fitness matrix $\Lambda =(\lambda _i^j)_{i,j}$, and using vector notation. Usually in the classical replicator equation, $\Theta =1$ because time is scaled arbitrarily. In contrast, in our derived replicator equation here, we have an explicit ‘clock’ for the frequency dynamics, set by the constant $\Theta$ (Table 1). The tempo of multi-strain “motion” on the slow timescale toward an equilibrium is determined by this pre-factor, which depends specifically on their absolute transmission rate $\beta$, but also nonlinearly on mean-field traits $R_0$ and *k*, via the conserved aggregated quantities ${T^*}$, ${I^*}$, ${D^*}$ (see Supplementary Figure S2). Note that $\Theta$ can be rescaled arbitrarily up to a multiplicative constant, factored out of matrix $\Lambda$, shifting the effective speed of non-neutral dynamics between types in the system. The second critical constant $\mu =\frac{{I^*}}{{D^*}}=\frac{1}{k(R_0-1)}$, in this replicator equation (within the $\Lambda$ entries), represents the ratio between single- and co-colonization prevalence in the neutral system, and is a crucial factor that amplifies the net effect of asymmetry in co-colonization interactions in the system, and consequently, the dynamic complexity of multiple strain frequencies (Gjini and Madec 2020).

The other key quantity in the *N*-strain frequency dynamics (13) is the quadratic term $Q(\tau )=\mathbf{z }^t \Lambda \mathbf{z }=\mathop {\sum \sum _{\kappa <j}}\lambda _j^{\kappa } z_j(\tau )z_{\kappa }(\tau )$, which couples all individual strain fitnesses. Indeed, if we define the *mean invasion fitness of strain j*, by summing over his relative ‘success’ on any other member:

$$\begin{aligned} \bar{\lambda _j}(\tau )=\sum _{\kappa \ne j} \lambda _j^{\kappa } z_{\kappa }(\tau ), \end{aligned}$$

then *Q* reads:

14 $$\begin{aligned} Q(\tau )=\sum _{j=1}^N \bar{\lambda _j}(\tau ) z_j (\tau ). \end{aligned}$$

Hence, this overall common feedback represents the *global mean fitness* of the system where the *dynamic* average is taken with respect to pairwise invasion. This sum over all extant pairs in the system reflects the mean ‘pairwise invasibility’ of the system as a whole, changing over time with strain frequencies $z_j(\tau ), z_{\kappa }(\tau )$. Upon closer inspection of (13), information on the resilience of a group of strains may be derived from the sign of *Q*: if $Q>0$ then each existing strain’s net growth is reduced within the group, but the overall community is more resistant to invasion by a new outsider strain, and viceversa, if $Q<0$, then each existing strain grows more within the group, but the overall community would inevitably be also more vulnerable to invasion by invader strains. The effect of *Q* on the frequency dynamics illustrates, in this system, the exact role of environmental feedback on eco-evolutionary processes (Lion 2018), highlighting the adaptive fitness landscape where selection in interaction trait space unfolds.

### Special Pairwise Invasion Structures Among *N* Strains

**Fig. 3:**
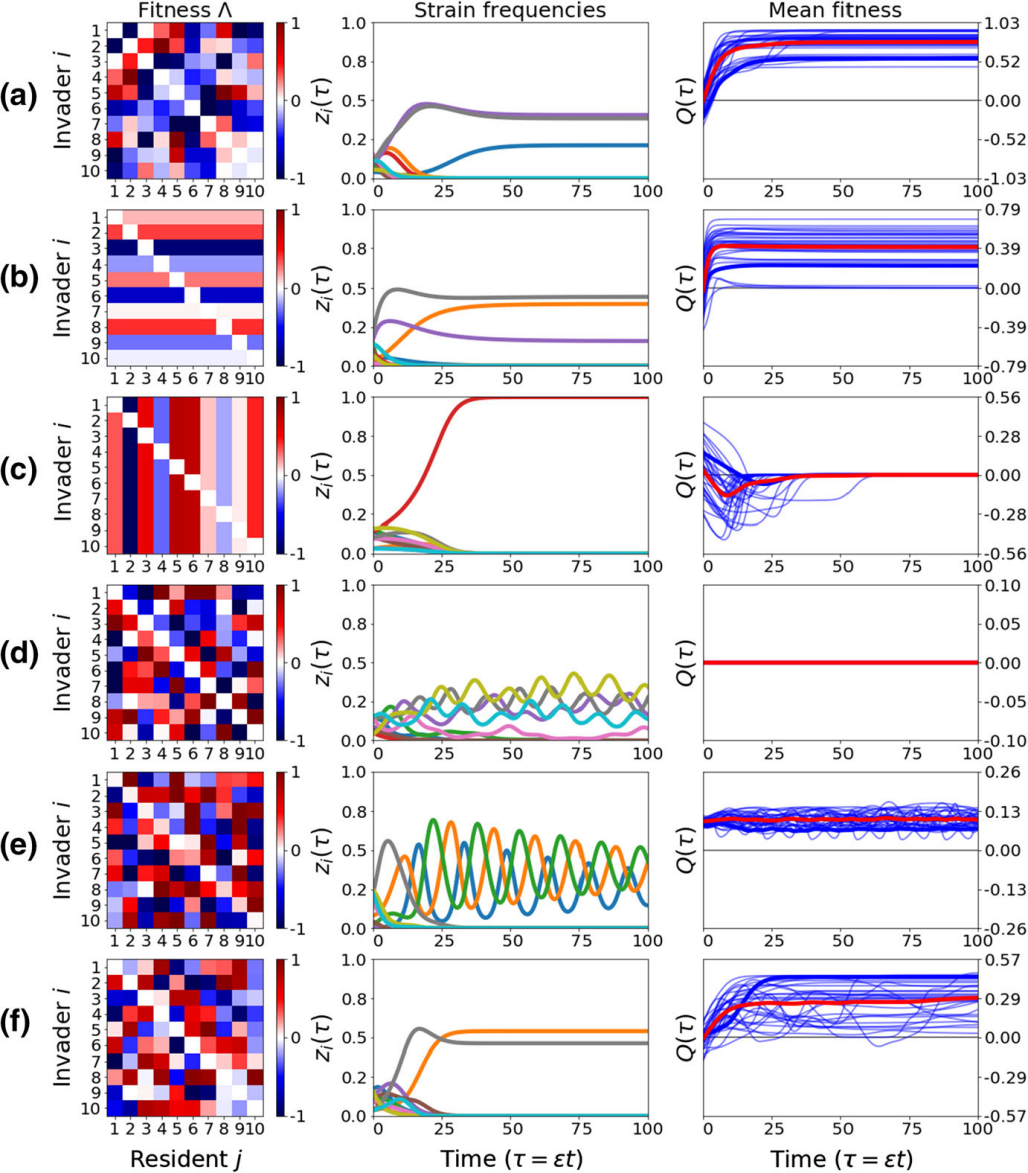
Canonical pairwise invasion structures ($\Lambda$) between *N* types and collective dynamics evolution. We generated random $\Lambda$ matrices, with $\lambda _{i}^j$ entries in the range $[-1,1]$, from 6 special cases, and simulated multi-type dynamics ($N=10$, $\Theta =1$) under many realizations of the model (13), starting from random initial conditions on the slow manifold, for each case. *Q* is the mean fitness term in the system (the common ‘environment’ for all types) changing differently depending on the pairwise invasion fitness matrix. In the third column, the thin blue lines indicate *Q* evolution for each realization, the thick blue line indicates *Q* evolution for the $z_i$ dynamics shown in the second column, and the thick red line depicts the mean over all 30 realizations. **a** Symmetric matrix. This corresponds to the same dynamics captured by Fisher’s fundamental theorem. **b** Invader-driven fitnesses (‘hierarchical attack’). Large potential for coexistence. **c** Resident-driven fitnesses (‘hierarchical defense’). Large potential for competitive exclusion. **d** Anti-symmetric invasion fitnesses. *Q* is exactly zero over all time and there is large potential for complex multi-strain behavior. **e** Almost-antisymmetric invasion fitnesses. Maintenance of potential for complex dynamics (e.g., limit cycles) leading to periodicity (but positivity) in *Q*. **f** Random mutual invasion. Rich model behavior is possible. On average coexistence is more likely, but increases as well as decreases in *Q* over a single realization are possible (Color figure online)

Next, we show how the uncovered model reduction can be used to gain deeper biological insight on the multi-strain co-colonization system. Special structures of co-colonization interactions *K* typically yield one of the canonical cases of the invasion fitness matrix $\Lambda$ (Table 2), which are easier to understand analytically with our replicator equation (13). We thus consider a few special cases for the invasion fitness matrix between strains, leading to special collective dynamics and mean fitness of the system (see Supplementary Material 4). Special cases of *Q* in $\lambda _{i}^{j}$ space are more straightforward to analyze than special cases in $K_{ij}$ trait space, because for each $\lambda _{i}^{j}$ representation (see Eq. 12), there is an infinite set of co-colonization interaction *K* matrices, leading to the same pairwise invasion network between strains. The following special invasion structures are illustrated in Fig. 3:

**Table 2:** Link between the structure of the co-colonization interaction matrix *K* and the pairwise invasion fitness matrix $\Lambda$

Co-colonization interaction matrix $K=(K_{ij})$	Pairwise invasion fitness matrix $\Lambda =(\lambda _i^j)$
$\bullet$Symmetric (general)	$\bullet$ General
$\quad K_{ij}=K_{ji}$	$\quad \lambda _i^j=\alpha _{ji}-\alpha _{jj}$
$\bullet$Symmetric (special 1)	$\bullet$Symmetric
$\quad K_{ij}=K_{ji}$ and $K_{ii}=k$	$\quad \lambda _{i}^j=\lambda _j^i=\alpha _{ij}$
$\bullet$Symmetric (special 2)	$\bullet$Invader-driven
$\quad K_{ij}=K_{ji}=K_{ii}+K_{jj}-k$	$\quad \lambda _{i}^j=\alpha _{ii}$
$\bullet$Symmetric (diagonal)	$\bullet$Resident-driven
$\quad K_{ij}=k$ if $i\ne j$	$\quad \lambda _{i}^j=-\alpha _{jj}$
$\bullet$Colonizer-driven	$\bullet$Anti-symmetric
$\quad K_{ij}=k_i$	$\quad \lambda _i^j=-\lambda _j^i=\mu (\alpha _j-\alpha _i)$
$\bullet$Cocolonizer-driven	$\bullet$Anti-symmetric
$\quad K_{ij}=k_j$	$\quad \lambda _i^j=-\lambda _j^i=(\mu +1)(\alpha _i-\alpha _j)$
$\bullet$Anti-symmetric	$\bullet$ Anti-symmetric
$\quad \frac{1}{2}\left( K_{ij}+K_{ji}\right) =k$	$\quad \lambda _i^j=-\lambda _j^i=2 \mu \alpha _{ji}$

Special structures of *K* yield one of the canonical cases of $\Lambda$, and thus relate to different types of multi-strain dynamics (see Fig. 3). Recall that for co-colonization interaction we have $K_{ij}=k+\varepsilon \alpha _{ij},$ and for pairwise invasion fitness we have $\lambda _i^j=\mu (\alpha _{ji}-\alpha _{ij})+\alpha _{ji}-\alpha _{jj},$ where $\mu =I^*/D^*$ is the single to co-colonization prevalence ratio. This formula may be inverted as: $\alpha _{ij}=\frac{\mu }{2\mu +1} \left( \lambda _i^j+\alpha _{ii}\right) + \frac{\mu +1}{2\mu +1} \left( \lambda _j^i+\alpha _{jj}\right)$. Note that a given matrix $\Lambda$—and then a given dynamics—is reached by an infinite set of matrices *K*

i. *Symmetric matrix* A symmetric $\lambda _{i}^{j}$ structure between strains in mutual invasion leads to a general feature of the dynamics whereby *Q* always increases over time (Fig. 3a). This case, namely the replicator equation for doubly symmetric games, is formally equivalent to the continuous time model of natural selection at a single (diploid) locus with *N* alleles, known as Fisher’s fundamental theorem of natural selection (Fisher 1958; Price 1972; Edwards 1994). In this case, it can be shown that the population mean fitness increases over time, with the rate of change in mean fitness equal to the trait variance at any point (see S4 for full verification of this feature also in our model). In our context, where $\lambda _{i}^j$ denote pairwise invasion fitness between any two strains, the increase in mean fitness during selective dynamics among *N* strains, implies that when pairwise invasion ‘games’ are symmetric, the system becomes more resistant to invasion by outsider strains over time. And this is a robust mathematical property, conserved also when the system is close to this case.
ii *Invader-driven invasion* In this case, columns of $\Lambda$ are equal, meaning it’s differences in ‘attack rates’ (invasiveness) of types that are defining their hierarchical dynamics (Fig. 3b). Mean fitness *Q* again evolves over slow time, reflecting the selection occurring in the multi-type system, and again tends to increase toward positive values, suggesting coexistence is more likely, although in special cases competitive exclusion may occur.
iii. *Resident-driven invasion* In this case, multi-strain dynamics are driven by variation in ‘defense’ or invasability (rows of $\Lambda$ are equal), and the principle of competitive exclusion (with possible multi-stability) applies more often, whereby the weakest strains are excluded, and only the best ‘defender’ of its territory (equilibrium when alone) survives. Competitive exclusion obviously implies *Q* should tend to 0, verified in Fig. 3c. In exceptional cases, coexistence may also be possible in this case (see S4 for details).
iv. *Antisymmetric matrix* This is the case when $\lambda _{i}^{j} = -\lambda _{j}^i$ and the propensity for complex coexistence dynamics between strains is very high (Fig. 3d). *Q* is exactly zero in this case, corresponding to zero-sum-games in evolutionary game theory (Hofbauer and Sigmund 2003). Like in the classical prey-predator Lotka-Volterra system, there exists a unique center surrounded by a family of cycles (Chawanya and Tokita 2002). This type of oscillatory coexistence between multiple strains is structurally unstable.
v. *Almost-antisymmetric* In this case, a small perturbation of the pure anti-symmetric structure in mutual invasion fitnesses disrupts the center leading to a stable or an unstable node. This gives rise to positive and periodic *Q*, where limit-cycles, heteroclinic cycles or chaos are more likely for multi-strain coexistence.
vi. *Random mutual invasion* In this case, which is the most general case, captured by our framework, the dynamics of *Q*(*z*) can be arbitrary, and increase or decrease over the same realization of multi-strain dynamics, thus encapsulating dynamic shifts in ’environment quality’, and unpredictable emergent dynamics of mean fitness over time (Fig. 3e).

While an exhaustive exploration of all possible structures falls beyond the scope of this paper, the rules of thumb outlined above, linking co-colonization interactions *K* with pairwise invasion fitness structures $\Lambda$, and system dynamics (Table 2, Fig. 3) can be a useful starting point for deeper biological investigation of particular host-microbe or interaction network scenarios.

For example, when co-colonization coefficients $K_{ij}$ display a row-wise or column-wise structure (see Lipsitch et al. (2012) for such hypotheses in pneumococcus), invoking a strain-specific definition of this trait, for $N=2$ the principle of competitive exclusion applies, but for general number of strains *N*, such special case of our model, where each edge of the network denotes competitive exclusion, collapses to the $Q=0$ case (antisymmetric invasion matrix above) and complex coexistence dynamics, of an odd number of strains, are expected (Chawanya and Tokita 2002). In practice, perfect identity across all rows/columns of *K* is too strict a criterion, and in reality, any small deviation from such extreme scenario should lead to an almost anti-symmetric pairwise invasion fitness structure.

### From Explicit Strain Dynamics Back to Epidemiological Variables

Next, we link strain frequencies back to the original epidemiological system with *N* strains, given by the SIS model with co-colonization interactions (1), assuming the $K_{ij}$ and the global epidemiological parameters are known. The key framing $K_{ij}=k+\varepsilon \alpha _{ij}$, needed for the model reduction, is mathematically non-unique, and can be applied with respect to any reference *k*, provided that the resulting $\varepsilon$ is small. However, one convenient choice is to define *k* as the average of the original co-colonization matrix entries $K_{ij}$:

15 $$\begin{aligned} k=\frac{\sum _{i,j} K_{ij}}{N^2}, \end{aligned}$$

and to define $\varepsilon$, as the root mean square distance of each $K_{ij}$ from their mean *k*:

16 $$\begin{aligned} \varepsilon =\sqrt{\frac{\sum _{i,j} (K_{ij}-k)^2}{N^2}}, \end{aligned}$$

thus representing the standard deviation of the $K_{ij}$ traits in the pool of *N* available strains. The direction of deviation from neutrality (bias) for the interaction between strain *i* and *j* is then obtained as:

17 $$\begin{aligned} \alpha _{ij}=\frac{K_{ij}-k}{\varepsilon }, \end{aligned}$$

whereby $$A=\begin{pmatrix} \alpha _{ij} \end{pmatrix}_{1\le i,j \le N}$$ is the *normalized interaction matrix*, with $\Vert A\Vert _2=\sqrt{\sum _{i,j} \alpha _{ij}^2} =N.$ This matrix *A*, and the ratio $\mu$, determine the pairwise invasion fitness matrix (Eq. 12), which then drive the non-neutral dynamics (Eq. 13). Provided $\varepsilon$ is small, the behavior of our approximation (10) describes very well the long-term dynamics of the original system (1). To recover the original variables from our approximation, we have a ‘conservation law’, reminiscent of other conservation laws in ecology (Hubbell 2001), for global quantities:

18 $$\begin{aligned} S(t)={S^*}:=\frac{m}{\beta }=\frac{1}{R_0},\quad T(t)={T^*}:=1-{S^*}=1-\frac{1}{R_0},\nonumber \\ \quad {\displaystyle {I^*}(t):=\frac{m {T^*}}{m+\beta k {T^*}}=\frac{ {T^*}}{1+R_0 k {T^*}}}, \quad {D^*}(t)={T^*}(t)-{I^*}(t) \end{aligned}$$

namely, the total prevalence of uncolonized hosts *S*, total prevalence of colonized hosts *T*, and respective prevalences of single and dual colonization, *I* and *D*. Further, to obtain strain-specific single colonization, and co-colonization prevalences in the system, we find:

19 $$\begin{aligned} I_i(t):= {I^*}z_i( \tau ),\qquad {\displaystyle I_{ij}(t)={D^*}z_i(\tau ) z_j(\tau )}. \end{aligned}$$

where the slow time scale is $\tau =\varepsilon t$, and the strain frequencies $z_i(\tau )$ verify $\sum _i z_i(\tau )= 1$ and follow explicit dynamics (13) (for details see also Table 1).

**Fig. 4:**
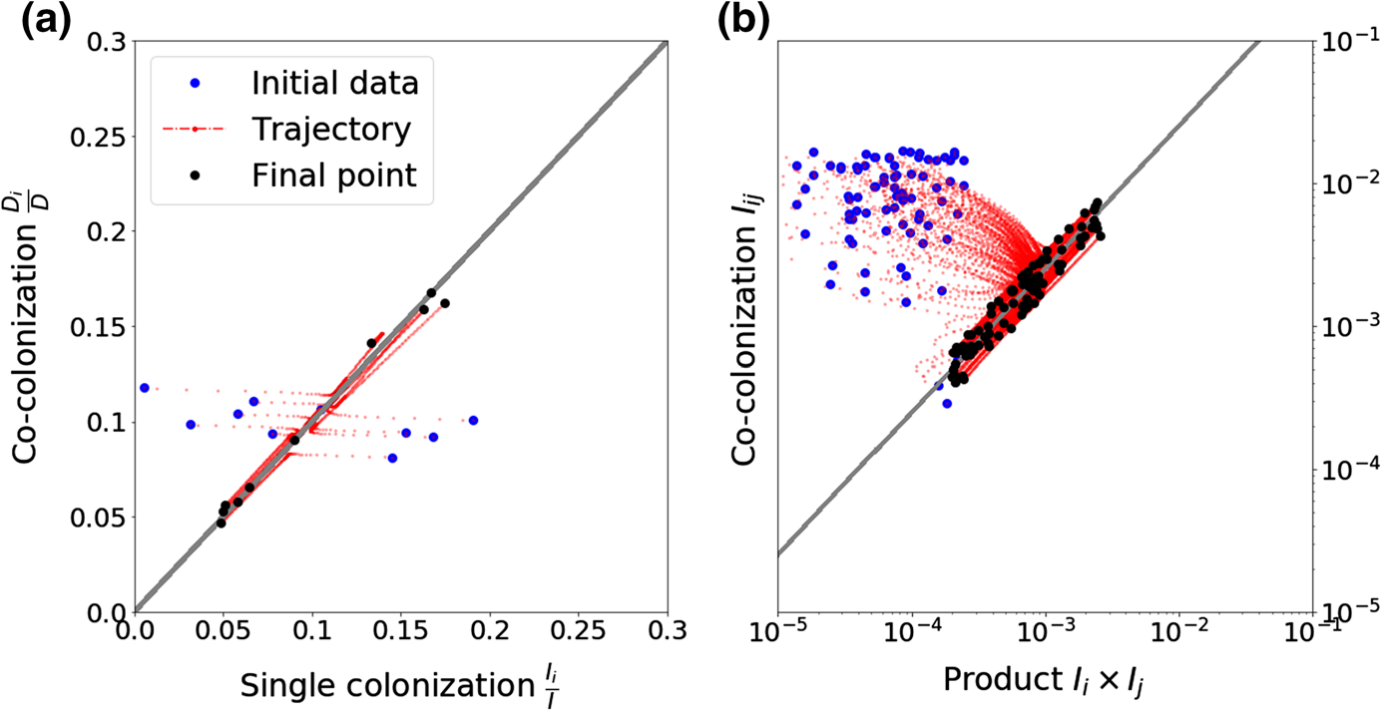
Illustration of invariant principles for strain coexistence on the long timescale. **a** Strain frequencies tend to equalize in single and co-colonization, for all strains and for all time when reaching the slow manifold (9). **b** Prevalence of co-colonization $I_{ij}$ tends to scale with the product of strain prevalences in single colonization ($I_i$, $I_j$), for all strain pairs and for all time, during the slow dynamics (Table 1). This example is simulated using a random matrix *K*, with $N=10$. Each trajectory corresponds to a given strain in the system (**a**), or a given strain pair (**b**). An example for $N=20$ is shown in Supplementary figure S3 (Color figure online)

The reconstitution of the epidemiological variables from the replicator equation exposes two special features of the multi-strain dynamics: (i) the $z_i$ variables, describing relative strain frequencies in the host population, tend to necessarily equalize in single and co-colonization (Fig. 4a); (ii) the prevalence of co-colonization with strains *i* and *j* (Fig. 4b), is proportional to the product between single prevalences of *i* and *j* in the population ($I_{ij} \sim I_i I_j$), thus confirming the $I_{ij}$ and $I_{ji}$ equivalence in this model. These two quasi-neutrality principles, are preserved on the slow timescale, independently of strain identities and for all time, and moreover, independently of dynamic complexity. Thus, these two features can be used as a quasi-neutrality test for high-dimensional interacting systems when multi-strain prevalence data are available.

#### Interpreting the Prevalence of Mixed Co-colonization

The fact that prevalence of co-colonization with strains *i* and *j* in this system, involves a product between single prevalences of *i* and *j* in the population ($I_{ij} \sim I_i I_j$), even though the strains are interacting, is contrary to the independence closure assumption under a purely statistical perspective, adopted heuristically in epidemic models (Kucharski et al. 2016). As we show here, depending on epidemiological details, the feedbacks between interacting strains may mathematically lead to overall multiplicative effects between individual and dual strain prevalences in colonization, with an explicit pre-factor determined nonlinearly by $R_0$ and *k*:

20 $$\begin{aligned} I_{ij}=kR_0[1+k(R_0-1)] I_i I_j. \end{aligned}$$

In particular, empirical co-occurrence of two strains less than expected by chance ($I_{ij}<I_{i}I_{j}$) is always an indicator of average competitive interactions in co-colonization, namely $k<1$. However, empirical co-occurrence of two strains more than expected by chance ($I_{ij}>I_{i}I_{j}$) can be an indicator of competition or cooperation in co-colonization, depending on $R_0$: if $R_0$ is small (low overall prevalence), such phenomenon would be compatible with mean cooperation ($k>1$), whereas if $R_0$ is large (high overall prevalence), such phenomenon would be compatible with competition in co-colonization ($k<1$), lending support to context dependence in the inference of strain interactions (Coyte and Rakoff-Nahoum 2019).

#### Accuracy and Computation Efficiency of the Approximation

Finally, to test the quality of the slow-fast approximation with respect to the original system, we verify that the error between the two is small. This is made precise via numerical simulations (Figure S4), where the neutral model is shown to be a good approximation of the original system in a fast time-scale, $o(1/\varepsilon )$, and the slow-dynamics reduction a good approximation on the longer time-scale ($\varepsilon t$). Numerically, we find that the approximation remains valid even for values of $\varepsilon$ that are not too small (e.g., $\varepsilon \in (0.1,0.3)$). Using the fast-slow decomposition for this model is also advantageous in terms of efficient computation of dynamics for an arbitrary number of strains *N* (Figures S5–S6). For example, when increasing the number of strains from 2 to 50, the average time it takes to compute dynamics in the original system increases from 45 sec. to 5 min, whereas using the slow dynamics approximation, the time of computation increases only from $10^{-2}$ to 0.5 sec. While reinforcing the validity of our method, the quality and speed of this model reduction are two important features could aid parameter inference frameworks for epidemiological time-series in high-dimensional multi-strain systems (Shrestha et al. 2011; Gjini et al. 2016).

Overall this multi-strain model, the slow-fast decomposition, and the key features outlined above provide a crucial foundation for the full characterization of conservative multi-strain SIS dynamics with interactions in co-colonization. Our conceptual and analytical framework emphasize that a closer integration between different temporal scales on one hand, and demographic vs. selective processes on the other, is possible for understanding multi-type communities.

## Discussion

Recent approaches in theoretical epidemiology are increasingly addressing the eco-evolutionary feedbacks between infectious disease dynamics and the diversity of co-circulating strains (Gog and Grenfell 2002; Day and Gandon 2006; Berngruber et al. 2013). In this study, we have linked epidemiology and evolution in a new context, by considering an endemic multi-strain system with diversity encoded exclusively in pairwise interactions upon coinfection and altered susceptibilities to coinfection between strains. Starting from an SIS compartmental model with singly and co-infected hosts (and no virulence), through a timescale separation, we obtained a model reduction, which maps explicitly the variation in pairwise strain interactions ($K_{ij}$) to a closed replicator equation (Eq. 10) governing strain frequency dynamics. We investigated how co-colonization interaction coefficients, be they cooperative or competitive on average (*k* below/above 1) and with arbitrary among-strain variation ($\alpha _{ij}$), drive coexistence in a system with *N* similar types. We find that it is not whether strains compete or cooperate in co-colonization that defines their success, but rather how much, and in which way, their mutual and polarized competition or cooperation deviates from the mean, and how this is modulated by the dynamism of the system.

The classical replicator equation in evolutionary game theory has a long history of study (Taylor and Jonker 1978; Weibull 1997; Hofbauer and Sigmund 2003). Here, however, in contrast to assuming it heuristically *a priori* (e.g., (Allesina and Levine 2011)), we have derived it from basic aggregation and timescale principles in an explicit biological context. The replicator equation is mathematically related to Lotka-Volterra systems (Lotka 1926; Volterra 1926), namely the continuous replicator equation on *N* types is topologically equivalent to the generalized Lotka–Volterra equation in $N-1$ dimensions (Bomze 1983, 1995; Hofbauer and Sigmund 2003). Thus, by uncovering replicator dynamics at the heart of our multi-strain SIS system with co-colonization, we offer new avenues of methodological and theoretical cross-fertilization between ecology, epidemiology and evolution.

### From Pairwise Invasion to Collective Coexistence

We have demonstrated that the collective dynamics of the *N*-strain system can be expressed entirely in terms of mutual invasion fitnesses of each pair of strains (Eq. 13). This is a novel and important finding that links mathematically pairwise outcomes to emergent community dynamics. Our results suggest that a bottom-up approach can be applied to understand and exactly predict community structure. In a recent experimental study, investigating assembly rules in microbial communities (Friedman et al. 2017), survival in three-species competitions was predicted by the pairwise outcomes with an accuracy of  90%. Yet, a similar level of accuracy in competitions between sets of seven or all eight species was harder to obtain, and required additional information regarding the outcomes of the three-species competitions. Despite their use of the generalized Lotka-Volterra framework, and our use of a multi-type SIS model, the key to obtain *N*-dimensional dynamics may be in exploiting the full nonlinear coupling between pairwise invasion fitnesses, made explicit here, in the replicator equation. For example, empirical measurement of $\lambda _i^j$ from pairwise invasion experiments among similar species may be fed into the *N*-dimensional replicator equation to anticipate their collective coexistence dynamics *in silico*. It would be straightforward to then test model predictions with the actual multi-species experiments. A key quantitative feature of our framework is that ‘edges’ between any two species in the network are not just resolved in terms of final outcome (coexistence, bistability, exclusion) but in terms of the actual magnitude of the initial growth rate during invasion, which holds more subtle information.

### Environmental Feedback from Higher-Order Interactions

Higher-order interactions are expected to emerge whenever the presence of an additional species changes the interaction between two existing species, and can impact on the maintenance of diversity (Billick and Case 1994). In our co-colonization system, modeling explicitly strain interactions with two types of resources: susceptibles *S* and singly colonized hosts *I*, a certain type of higher-order interactions arise naturally because of the indirect effects that altered susceptibilities between any pair *i* and *j* in co-colonization have on suppressing or augmenting the available resources $I_i$ and $I_j$ for the rest of the community, and thus when summed, contribute to mean fitness among everybody in the system. In this entangled network, the multiple types modulate their common environment through the changing term *Q* in (13), which can mean ‘deterioration’ of the environment if $Q>0$ or ‘amelioration’ of the environment if $Q<0$. This does not necessarily imply that strains become more cooperative or competitive in epidemiological co-colonization, as the dynamics of the mean susceptibility to co-cocolonization at the single host level, depending on *q* (in Eq. 10), can be different from the dynamics of mean fitness *Q* at the level of the strain system.

Our expression for strain frequency evolution (13) makes it also explicit that ‘environmental deterioration’ may be seen as a cost for the existing collective (since it reduces each strain’s rate of growth), but it serves as a protective mechanism against invasion by an outsider strain, and viceversa: ‘amelioration’ may on one hand seem like it benefits all strains, but on the other it also benefits any outsiders, which eventually may invade more easily. Central to these insights is having made explicit in this particular model the dependence on environmental dynamics of the selective dynamics between types (Lion 2018), both in invasion fitness trait space ($\lambda _{i}^j$), and in cocolonization trait space ($\alpha _{ij}$). How strain diversity exactly shapes the system property *Q* (Hooper et al. 2005), and how *Q* in turn shapes diversity remains to be studied in the future.

### Invariant Principles in *N*-Type Co-colonization

As known from frequency-dependent selection in evolutionary games, the final outcome among *N* players can be complex, represent a non-fitness-maximizing equilibrium and include oscillations and chaos (Nowak and Sigmund 2004; Cressman and Tao 2014). Yet, here we find invariant principles emerging in non-equilibrium multi-type dynamics: The first one being about the dominance of types in single and co-colonization, which is expected to be equal, and the second one being about the co-colonization prevalences as a function of single colonization prevalences of strains (Figure 4, Supplementary Figure S6). These could be used as a practical test for quasi-neutrality, when strain prevalence data are available. Our recapitulation of co-colonization dynamics from strain frequencies sheds new analytical light on pathogen interactions and their epidemiological manifestation (Kucharski et al. 2016), providing the link between within-host co-occurrence and population-level prevalences of strains. While independence underpins a majority of methods for detecting pathogen interactions from cross-sectional survey data ( e.g., Valente et al. 2012; Cobey and Lipsitch 2012b), it is being recognized that even simple epidemiological models challenge the underlying assumption of statistical independence (Hamelin et al. 2019). Studies are showing that even if pathogens do not interact, other epidemiological feedbacks can induce positive correlation between their prevalences, which leads the proportion of co-infected hosts to be higher than multiplication would suggest.

Along similar logic, our results clearly expose that even if pathogens interact (e.g., via altered susceptibilities to coinfection), multiplicative effects between their prevalences emerge in co-colonization, but with an explicit pre-factor dependent on overall transmission and mean interaction coefficient. This invites a revision of methods to identify interactions between pathogens in endemic systems from cross-sectional data, based on a deeper mathematical understanding of underlying feedbacks and context-dependence (Coyte and Rakoff-Nahoum 2019).

### Extensions and Outlook

In our system, at most two microbial strains can concurrently infect a host. Extension to higher multiplicity of infection (MOI) could be of interest in the future, starting from the current setup, or taking advantage of previous frameworks (Adler and Brunet 1991). The transmission and clearance rates of all strains in our model were assumed equal, unlike other multi-strain SIS models (Thieme 2007; Martcheva 2009; Bichara et al. 2014), because our aim was to focus on coinfection, coexistence, and the pairwise interaction matrix between strains. In an ongoing work, we show that relaxing this assumption, within the same model structure, leads to the *same* replicator equation with invasion fitnesses. But each pairwise invasion fitness, in that case, becomes a combination of deviations from neutrality in all traits (unpublished). Accounting for asymmetry in other traits and coinfection requires us to specify in the model, besides overall transmission in singly and co-infected hosts (here assumed equal), additional features such as the transmission rate of each of the coinfecting strains in mixed infection classes and their clearance rate. It is likely that several model structures, or perturbations, branching out of the core and simple formulation analyzed in this paper, can lead to similar replicator equation-like dynamics in less dimensions, but this requires further mathematical investigation.

Past theoretical work has considered vulnerability to co-infection modeling it as a single mean-field parameter (Alizon 2013a). Others have studied how this trait at the host-pathogen interface impacts disease persistence (Gaivão et al. 2017), coexistence and vaccination effects (Lipsitch 1997; Gjini et al. 2016), and how it contributes to diversity in other traits, e.g., virulence (Alizon et al. 2013) and antibiotic resistance (Davies et al. 2019). With the here-proposed analytical framework, exploration of such processes, indirectly affected by co-colonization, could be enhanced and generalized to higher number of strains.

However, increasing structural complexity in the model is likely to increase the intrinsic dynamic complexity of the system, independently of the number of strains. For example, Gaivão et al. (2017) relax the assumption of equal clearance and transmission rates from single and dual colonization, and find the criteria under which these asymmetries enhance endemic persistence. By obtaining a backward bifurcation near $R_0 = 1$, they highlight that sufficiently higher reproductive value of the parasite in multiply-infected hosts can enable parasite persistence, and in such case, the mean-field susceptibility to co-colonization (*k*) gains a vital importance.

Sequential clearance of each strain from co-colonized hosts is also a relevant and important model extension, instead of the direct clearance assumed in our model. As noted also by Gjini et al. (2016) and Gjini and Madec (2017), this feature would cause the independence of total carriage from co-colonization parameters ($1-1/R_0$ here) to break down. This would give rise to bidirectional feedbacks between strain selection in co-colonization trait space and overall prevalence, which in the current framework only act in one direction: from total prevalence to strain selection via $\mu$ in $\lambda _{i}^j$ and the parameter $\Theta$ in Eq. (13).

Inevitably, the deterministic formulation adopted here does not allow to explore stochastic effects in selection, which in the quasi-neutral limit may become increasingly important (Constable and McKane 2018; Kogan et al. 2014). Noise-induced selection may happen during the fast (neutral) time-scale of the dynamics, where strains should behave as equivalent. This would be interesting for the future.

Even within our simplifying assumptions, there is a lot of complex dynamics for different *N* and different matrix structures that we have not addressed in this very model, including multistability, limit cycles and chaos. Yet, our results thus far open promising avenues. The wider and more complete ecological picture of co-colonization, as well as the gradients in diversity-stability regimes in coexistence are the focus of another study (Gjini and Madec 2020). A natural next step is harnessing more parallels with the classical Lotka–Volterra model (Lotka 1926; Volterra 1926; Mougi and Kondoh 2012; Friedman et al. 2017). Like for the Lotka–Volterra system, many mathematical results for general and special cases of the replicator equation already exist (Hofbauer and Sigmund 2003; Sandholm 2010; Cressman and Tao 2014), and these would carry over automatically in the multi-strain setting studied here. Importantly, the power of the replicator equation, which describes strain frequencies over time, lies in the explicit characterization of the mean fitness of the collective. Furthermore, the derivation we provide here has direct parameters coming from the biology of multi-strain colonization. This should enable clear translation to fitness (Metz et al. 1992) and expand the quantitative insights for multi-species competition beyond binary payoffs (Allesina and Levine 2011).

In summary, although motivated by infectious disease transmission with altered susceptibilities in co-colonization (Gjini et al. 2016; Gjini and Madec 2017), the global contagion dynamics captured here provide compelling parallels and invite applications in other systems. The coinfection model could be applied to study mechanistically coexistence in microbial consortia, plant ecology, opinion propagation dynamics, and other multi-type systems where colonizer-cocolonizer interactions matter. Thanks to its abstraction and simplicity, this model with its closed replicator equation for strain frequencies offers a new bridge between population dynamics in epidemiology, community ecology, and Darwinian evolution.

## Electronic supplementary material

Below is the link to the electronic supplementary material.

Supplementary material 1 (pdf 1020 KB)

Supplementary material 2 (mp4 276 KB)

Supplementary material 3 (mp4 1580 KB)

## Footnotes

1 While strictly speaking, infection elicits a specific immune response whereas colonization does not, typical SIS models for non-immunizing (usually bacterial) infections, still adopt the term infection, more as an epidemiological paradigm (Adler and Brunet 1991; Mosquera and Adler 1998; Alizon 2013a). Throughout the paper, for consistency, we will favor the term co-colonization, to highlight the avirulent and non-immunizing aspects of the infectious entities, but this *N* strain SIS co-infection model should apply in wider settings.
